# Differentiation of early germ cells from human skin-derived stem cells without exogenous gene integration

**DOI:** 10.1038/srep13822

**Published:** 2015-09-08

**Authors:** Wei Ge, Hua-Gang Ma, Shun-Feng Cheng, Yuan-Chao Sun, Li-Lan Sun, Xiao-Feng Sun, Lan Li, Paul Dyce, Julang Li, Qing-Hua Shi, Wei Shen

**Affiliations:** 1 Institute of Reproductive Sciences, Qingdao Agricultural University, Qingdao, Shandong 266109, China; 2 Key Laboratory of Animal Reproduction and Germplasm Enhancement in Universities of Shandong, College of Animal Science and Technology, Qingdao Agricultural University, Qingdao, Shandong 266109, China; 3 Reproductive Center, Weifang City People’s Hospital, Weifang, Shandong 261041, China; 4 Department of Animal and Poultry Science, University of Guelph, Guelph, Ontario N1G2W, Canada; 5 Molecular and Cell Genetics Laboratory, The CAS Key Laboratory of Innate Immunity and Chronic Disease, Hefei National Laboratory for Physical Sciences at Microscale, School of Life Sciences, University of Science and Technology of China, Hefei, Anhui 230027, China; 6 Collaborative Innovation Center of Genetics and Development, Fudan University, Shanghai 200433, China

## Abstract

Infertility has long been a difficult issue for many couples. The successful differentiation of germ cells and live progeny from pluripotent stem cells brings new hope to the couples suffering with infertility. Here we successfully isolated human fetus skin-derived stem cells (hfSDSCs) from fetus skin tissue and demonstrated that hfSDSCs can be differentiated into early human germ cell-like cells (hGCLCs). These cells express human germ cell markers DAZL and VASA. Moreover, these pluripotent stem cell-derived hGCLCs are free of exogenous gene integration. When hfSDSCs were differentiated in porcine follicle fluid (PFF) conditioned media, which has been shown to promote the differentiation of mouse and porcine SDSCs into oocyte-like cells (OLCs), we observed some vesicular structures formed from hfSDSCs. Moreover, when hfSDSCs were cultured with specific conditioned media, we observed punctate and elongated SCP3 staining foci, indicating the initiation of meiosis. Ploidy analysis and fluorescent *in situ* hybridization (FISH) analysis indicated that a small percentage of putative 1N populations formed from hfSDSCs when compared with positive controls. In conclusion, our data here, for the first time, demonstrated that hfSDSCs possess the differentiation potential into germ lines, and they may differentiate both male and female hGCLCs *in vitro* under appropriate conditions.

Recent studies demonstrated that adult human tissue-derived induced pluripotent stem (iPS) cells can be induced into human primordial germ cell-like cells (hPGCLCs) *in vitro*^1^,^2^,^3^. These hPGCLCs displayed similar characteristics to their *in vivo* counterparts in both gene expression and epigenetic status. Other studies also reported that iPS cells reprogrammed by human dermal fibroblasts have a robust ability to differentiate into hGCLCs via xenotransplantation into murine seminiferous tubules, these hGCLCs also show similar properties to *in vivo* human germ cells^4^,^5^. Together with live mouse offspring derived from mouse iPS cells, all previous studies demonstrated that iPS cells possess the intrinsic ability to differentiate into germ cells that can even give rise to live progeny^6^,^7^. The processes of iPS cell reprogramming require exogenous gene integration or other small-molecule compounds induction, however, public concerns on the application of iPS cells mainly focus on the tumorigenicity and immunogenicity of transplanted iPS cells^8^,^9^. There is an increasing need for the use of safer pluripotent stem cell types in reproductive medicine and therapeutic methods that are free of exogenous gene integration from the perspective of currently practical needs. Interestingly, skin-derived stem cells (SDSCs) from porcine or mouse also show the ability to give rise to germ cell-like cells (GCLCs) even without reprogramming into the iPS cell stage^10^,^11^,^12^. These SDSC-derived germ cells also express germ cell markers and show similar DNA methylation patterns to that of their *in vivo* counterparts^12^.

Derivation of germ cells or even live progeny *in vitro* provides an unparalleled platform for further studying mechanisms underlying gametogenesis, which is not accessible particularly in humans during early embryogenesis^13^. SDSCs, on the contrast, are more applicable when compared with iPS cells reprogrammed from adult tissues since tumorigenicity and immunogenicity of iPS cells transplanted have not been perfectly solved yet^8^,^9^. Noteworthy, SDSCs can be differentiated into neurons, astrocytes, and adipocytes *in vitro*^14^, demonstrating their multi-lineage differentiation potential. More importantly, SDSCs have been demonstrated to have germline potential by several previous studies and our unpublished data^10^,^11^,^12^,^15^. Here we demonstrated that hfSDSCs showed the potential for germline differentiation *in vitro*, which may provide a new model for elucidating mechanisms underlying human gametogenesis.

## Results

### Characterization, isolation and *in vitro* culture of hfSDSCs

**Figure 1:**
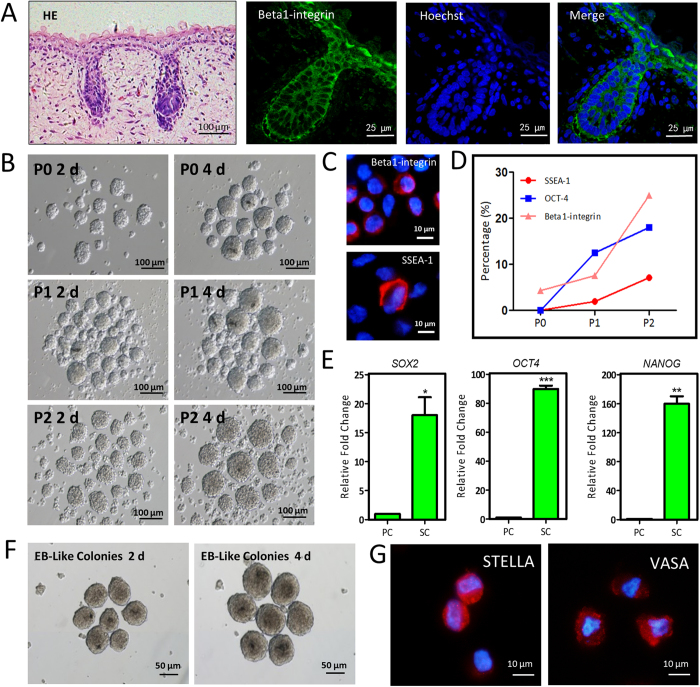
Identification and embryoid body (EB) differentiation of human fetus skin-derived stem cells (hfSDSCs). (**A**) Identification of human SDSCs from 4 months female skin tissue by antibody against Beta1-integrin. (**B**) hfSDSCs formed floating spheres when cultured *in vitro*. (**C**) Immunofluorescent staining of Beta1-integrin and SSEA-1 in hfSDSCs. (**D**) The percentage of OCT-4-, SSEA-1- and Beta1-integrin-positive hfSDSCs. (**E**) Expression of pluripotent markers *OCT4*, *SOX2* and *NANOG* in hfSDSCs by qRT-PCR. PC: primary culture, SC: sub-culture. (**F**) Typical EB-like colonies formed from hfSDSCs and these colonies are morphologically similar to EBs formed from human ESCs. (**G**) Germ cell markers VASA and Stella were found after EB induction for 4 days.

Human fetus skin tissues (3–5 months gestational age) were collected from a local hospital after elective abortion. The skin tissues were trypsinized with TypLE Express for 15–30 min at 37 °C according to the gestational age and hfSDSCs were harvested as previously described^10^. The location of hfSDSCs within the fetus skin tissue was illustrated by Hematoxylin/eosin (HE) staining and Beta1-integrin immunostaining (Fig. 1A). Obviously, hfSDSCs were located on the hair follicles structures and the surface of skin tissues. Similar to mouse and porcine SDSCs, hfSDSCs formed floating spheres when cultured *in vitro* (Fig. 1B). The positive signals of Βeta1-integrin and SSEA-1 (stage-specific embryonic antigen-1) were observed by immunofluorescence (Fig. 1C). When hfSDSCs were subcultured *in vitro*, the percentage of hfSDSCs that expressed the pluripotent markers was increased (Fig. 1D), and the mRNA expression of pluripotent markers including *OCT4* (*POU5F1*, POU Class 5 Homeobox 1), *NANOG* (Nanog Homeobox) and *SOX2* (Sex Determining Region Y-Box 2) was elevated (Fig. 1E). Karyotyping data revealed that *in vitro*-cultured hfSDSCs exhibited normal chromosome status (Supplementary Fig. S1A). Furthermore, the formation of embryoid body (EB) like structure was observed when hfSDSCs were cultured in EB induction media (Fig. 1F). Interestingly, EB-like colonies formed from hfSDSCs exhibited similar morphology as those formed from human ES cell lines. Immunofluorescence data also showed the expression of STELLA and VASA in the EB-like structures (Fig. 1G). Moreover, teratoma-like structures were observed when these EB-like colonies were further transplanted under the kidney capsule of SCID/Beige mice, which may explain their ability to give rise to GCLCs (Supplementary Fig. S1B).

### Derivation of human early germ cell-like cells by PFF conditioned media

**Figure 2:**
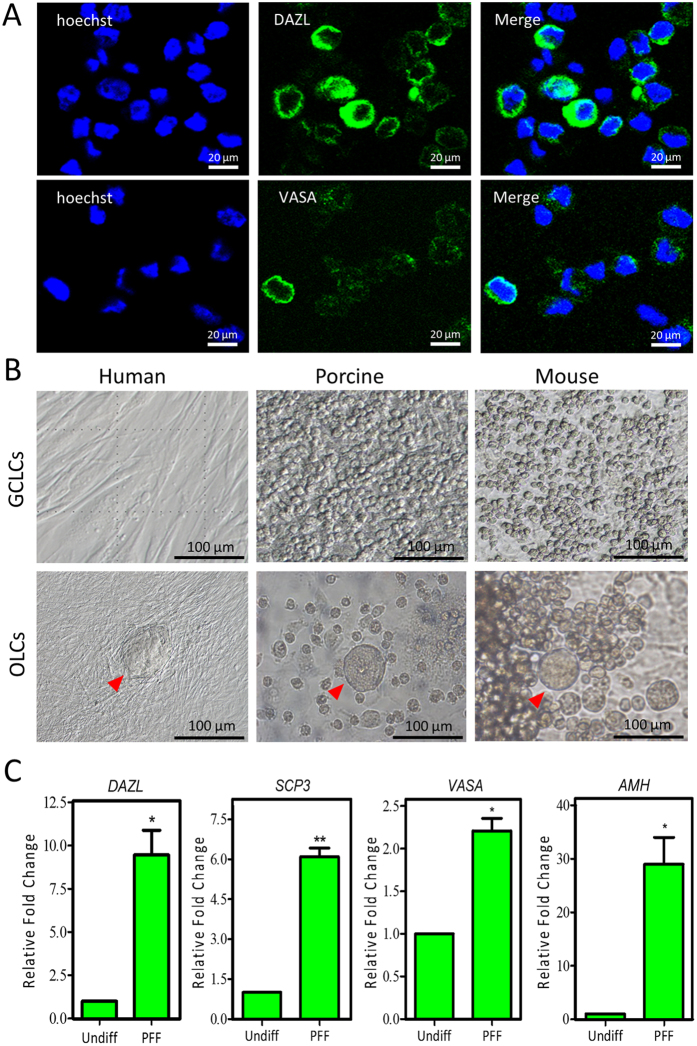
Differentiation of hfSDSCs into human germ cell-like cells (hGCLCs) in porcine follicle fluid (PFF) conditioned media. (**A**) DAZL- and VASA-positive hGCLCs. (**B**) Differentiation of human, porcine and mouse SDSCs in PFF conditioned media. (**C**) Expression of germ cell and meiosis markers including DAZL, VASA, SCP3 and AMH in hfSDSCs-differentiated cells using PFF conditioned media.

Since our previous study demonstrated that fetal porcine and newborn mouse SDSCs have the intrinsic ability to differentiate into GCLCs^10^,^11^,^12^, we next verified whether hfSDSCs can also be induced into hGCLCs *in vitro* by using the same protocol. Immunofluorescence data revealed that some subpopulations of the hfSDSCs exhibited positive expression of hGCLCs biomarkers including DAZL (Deleted in Azoospermia-like) and VASA (Fig. 2A). Previous study reported that shiny colonies of porcine or mouse GCLCs are morphologically distinctive after 15–30 days of differentiation^10^,^11^,^12^, interestingly, the hGCLCs did not show morphological distinction from other cells (Fig. 2B). hGCLCs aggregated with peripheral cells and cannot be distinguished from them via morphology. Although oocyte-like cells (OLCs) differentiated from SDSCs were observed in the mouse and porcine models, we did not observe typical OLCs using the same method. However, the vesicular structures were observed when hfSDSCs were differentiated with PFF conditioned media (indicated by red arrow) (Fig. 2B). Surprisingly, we unexpectedly observed that granulosa cell marker AMH (Anti-Mullerian Hormone) was upregulated in the vesicular structures, together with the elevated expression of germ cells markers and meiosis markers including DAZL, VASA and SCP3 (Synaptonemal Complex Protein 3) (Fig. 2C and Supplementary Fig. S2A). These indicate that these structures may be similar to the OLCs seen in the mouse and porcine models.

### Putative 1N population formation from hfSDSCs

**Figure 3:**
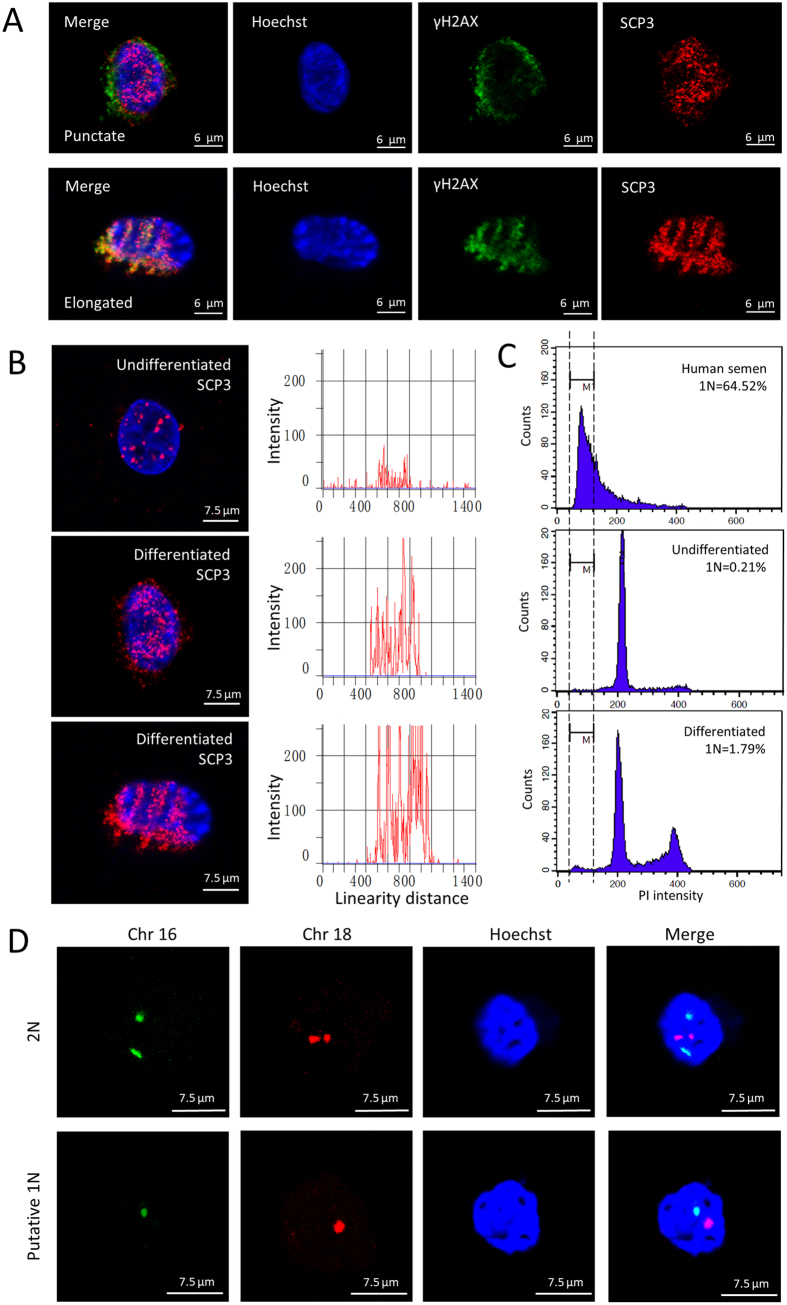
Putative haploids formation from hfSDSCs. (**A**) The spreading of synaptonemal complexs from hfSDSCs. both punctate and elongated staining patterns were observed. (**B**) Comparison of SCP3 staining intensity between differentiated and undifferentiated hfSDSCs. (**C**) Analysis of DNA content in differentiated and undifferentiated hfSDSCs. 1N cells indicate the formation of putative haploids. (**D**) Fluorescent *in situ* hybridization for chromosome 16 and 18 indicates that putative 1N populations formed from hfSDSCs.

The majority of previous studies focused on the differentiation potential of SDSCs into female GCLCs, here, we also explored whether these SDSCs (both XX and XY) have the intrinsic ability to differentiate into male GCLCs. We have compared several methods that have been successfully used in human embryonic stem cells (ESCs) differentiation into putative haploid cells. All of these methods are free of exogenous gene integration so no worry regarding iPS cells is involved here. Interestingly, after 18 days differentiation, one of differentiated groups showed a little higher percentage of putative 1N cells (Supplementary Table S1). The dynamics of the putative 1N population were tracked for a month via flow cytometry, and we observed the highest putative 1N population after 25 days of differentiation (Supplementary Fig. S2B). In our further investigation, the spreading of synaptonemal complexes was analyzed to determine the meiotic progression of hfSDSCs differentiation as previously described^2^, and both punctate and elongated staining patterns were observed (Fig. 3A). This result suggests that the initiation of meiosis may start but fail to fully elongate. Similar to human ESCs, undifferentiated hfSDSCs also showed punctate SCP3 foci, however, our further investigation indicated that differentiated hfSDSCs possessed more SCP3 foci compared with undifferentiated hfSDSCs (Fig. 3B). Furthermore, we observed a 1.79% of putative 1N population via flow cytometry after 25 days differentiation (Fig. 3C). It is worth mentioning that the differentiation of hfSDSCs into putative haploid cells did not show a rise in spermatogonial stem cell (SSC) conditioned media (Diff Media 1) while a higher percentage of putative haploid cell formation was observed in a differentiation media containing LIF (Leukemia Inhibitory Factor), FSH (Follicle-stimulating Hormone), EGF (epidermal growth factor), and ITS (Insulin Transferring Selenium) (Diff Media 2) (Supplementary Table S1), which is different from previous observations using human ESCs and mouse SSCs^16^,^17^. To further verify the putative 1N populations as indicated by FACs analyze, FISH probes against human chromosome 16 and 18 were used to analyze putative 1N populations and normal hfSDSCs (Fig. 3D), FISH data also indicates that putative haploids formed from hfSDSCs after differentiation. Ploidy analysis together with FISH analysis both demonstrated that putative haploids populations formed from hfSDSCs.

## Discussion

Derivation of GCLCs from SDSCs has been previously reported using model animals like porcine and mouse^10^,^11^. However, it is still unclear whether human SDSCs have the similar potential to differentiate into human early germ cells. Our data here demonstrated that hfSDSCs, similar to porcine and mouse models, can differentiate into human early GCLCs that express the same markers to their *in vivo* counterparts. Moreover, some vesicular structures were observed when hfSDSCs were differentiated in the same conditions as that in porcine and mouse models, and granulosa cell marker AMH was upregulated when hfSDSCs were differentiated in PFF conditioned media. These data support the differentiation potential of hfSDSCs into female germ lines. Furthermore, we demonstrated that hfSDSCs can differentiate into putative haploid cells with punctate and elongated SCP3 staining foci. These data demonstrate that hfSDSCs may also possess the differentiation potential into male germ lines *in vitro*. However, our human hfSDSCs are different from those in porcine and mouse models because we did not observe the formation of OLCs when hfSDSCs were differentiated in PFF conditioned media.

Although derivation of germ cells or even live progeny from stem cells has already been achieved in model animals such as mouse, public concerns still remain on these “viable” progeny^18^ because stem cells that can successfully differentiate into live progeny are currently restricted to ESCs and iPS cell populations. Therefore, the medical application of these two cell types is still a big challenge because many obstacles including ethical problem and safety concerns hinder their application^19^,^20^. Patient-derived stem cells without exogenous gene integration undoubtedly have unparalleled advantages in medical therapeutic applications when compared with ESCs and iPS cells.

Our previous investigations demonstrated that SDSCs from fetal porcine and newborn mouse can differentiate into early GCLCs that express the same markers as *in vivo* counterparts^10^,^11^,^12^. These early germ cells can further differentiate into OLCs that can restore estrus cycling in ovariectomized mice^21^. All these data indicate that SDSCs may be used as autologous stem cell types for medical applications. Interestingly, hfSDSCs cannot differentiate into OLCs according to our established protocol in porcine and mouse models. Moreover, early GCLCs from mouse and porcine are morphologically distinctive from surrounding cells characterized by shiny colonies. However, human early GCLCs are not morphologically distinctive, more specifically, they aggregate with surrounding cells and form vesicular structures after 2 weeks differentiation. Together with up-regulation of granulosa cell marker AMH, we speculate that hfSDSCs can differentiate into female germ cells. The derivation of human female germ cells from hfSDSCs requires more optimized differentiation conditions. One suggestion is to try human follicular fluid. If successful, the transplantation of these autologous OLCs may be used as a therapeutic method for restoring estradiol production in menopausal women as indicated by previous study on mouse model^21^.

Previous studies mainly focused on the differentiation potential of SDSCs into female germ line^10^,^11^,^12^. Here we also demonstrated hfSDSCs can differentiate into male GCLCs. We compared several differentiation methods that were used previously in human ESCs and iPS cells^22^,^23^,^24^,^25^. Surprisingly, we observed that one of our differentiation methods showed an increased population of putative 1N cells using DNA content analysis and FISH analysis, which are the indicatives for the formation of putative haploid cells and the differentiation potential of hfSDSCs into male germ line. However, we did not investigate whether these putative 1N populations were fertilizable, which were, in fact, the benchmark for *in vitro* derived haploids from human pluripotent stem cells.

In conclusion, this study demonstrated the differentiation potential of hfSDSCs into germ lines that express the same biomarkers as their *in vivo* counterparts. Moreover, hfSDSCs can differentiate into female and male GCLCs under appropriate conditions.

## Materials and Methods

### Isolation and *in vitro* culture of hfSDSCs

Human fetal skin tissues were collected from Weifang City People’s Hospital. The informed written consent was obtained from all patients in accordance with national guidelines and this study was approved by the Ethics Committee of Weifang City People’s Hospital and the Ethical Committee of Qingdao Agricultural University (Agreement No. 2013-16). Total 25 experiment samples (18 XX, 7 XY) were used in this study. hfSDSCs were isolated as previously described with some modifications^10^. Briefly, human skin tissues were washed three times with DMEM/F12 (1:1) basic (Gibco, Beijing, China) supplemented with 100 U/ml penicillin and 100 mg/ml streptomycin (HyClone, Beijing, China). The skin samples were cut into small pieces (<1 mm^2^) with scissors, trypsinized with TypLE Express (Gibco) for 15–30 min according to the gestational age. The typsinized skin tissues were washed three times with DMEM/F12 (1:1) basic and mechanically dissociated with a 1 ml pipette tip. Cell suspensions were then filtered with a 40 μm cell strainer (Corning, New York, USA). Cell pellets were seeded in a suspension tissue culture dish (SARSTEDT, Newton, USA) and the cells were passaged every 3-4 days. hfSDSCs were maintained in hfSDSCs media containing DMEM/F12 (1:1) basic (Gibco), 2% B27 supplement (Gibco), 20 ng/ml EGF (Sigma, St Louis, USA), 40 ng/ml recombinant human FGF-basic (bFGF, PeproTech, Rocky Hill, USA), penicillin (100 U/ml) and streptomycin (100 mg/ml).

### RNA isolation and RT-PCR

Total RNA was extracted using RNAprep pure MicroKit (Aidlab, Beijing, China) following the manufacturer’s instructions. cDNA was synthesized by using cDNA Synthesis Kit (TaKaRa, Dalian, China). Reverse transcription parameters were set as follows: 42 °C for 50 min and 65 °C for 15 min. For PCR, the primers were listed in table 1. PCR amplification was carried out on a Light Cycler real-time PCR instrument (Roche, Mannheim, Germany). The amplification procedure was conducted according to manufacturer’s instructions using a Light Cycler SYBR Green I Master (Roche). The PCR parameter were set at 95 °C for 10 min, followed by 55 cycles at 95 °C for 10 s, 60 °C for 30 s, and finally cooling at 4 °C.

### Porcine follicle fluid (PFF) preparation

Porcine ovaries were collected from a local slaughter house and washed with saline containing 50 U/ml penicillin and 50 mg/ml streptomycin. PFF was collected with a 10 ml syringe from ovarian follicles with diameter less than 3 mm. PFF was centrifuged twice at 3500 rpm for 10 min at 4 °C and the supernatant was filtered with 0.45 μm (Pall Corporation, Ann Arbor, USA) and 0.22 μm (Millipore, Carrigtwohill, Ireland) filter membranes. PFF was stored at −20 °C until use.

### hfSDSCs differentiation

For derivation of hGCLCs from hfSDSCs, hfSDSCs were differentiated as previously described^11^. Briefly, purified hfSDSCs were trypsinized with TypLE Express (Gibco), then cell pellets were seeded in a 24 well suspension dish. EB differentiation medium consisting of TCM 199 (Gibco), supplemented with 3 mg/ml BSA (Sigma), 1 mg/ml fetuin (Sigma), 5 μl/ml insulin transferring selenium (ITS, Gibco), 0.23 mM pyruvic acid (Gibco), 1 ng/ml EGF (Epidermal Growth Factor, Sigma), 20 ng/ml Activin A (Sigma), and 30 ng/ml BMP4 (R&D systems, Minneapolis, USA) was added for 4 days of differentiation and changed every two days. For derivation of OLCs from hfSDSCs, cells were seeded in a 6 cm dish with OLCs differentiation medium containing DMEM/High glucose (Gibco) supplemented with 5% FBS (Gibco), 5% filtered porcine follicular fluid (See PFF preparation procedure), 0.23 mM sodium pyruvate (Hyclone), 2 mM L-glutamine (Gibco), 0.1 mM non-essential amino acids (NEAA, Gibco), and 0.1 mM β-mercaptoethanol (Amresco, Beijing, China), penicillin (100 U/ml), streptomycin (100 mg/ml). For derivation of putative haploid cells from hfSDSCs, cells were differentiated in Diff medium 2 containing DMEM: F12 (1:1) basic (Gibco), supplemented with 15% FBS (Fetal Bovine Serum, Gibco), 1500 U/ml LIF (Sigma), 50 international units (mIU) FSH (Sigma), 10 ng/ml EGF (Sigma), 2% B27 supplement (Gibco), 5 μl/ml ITS (Gibco), penicillin (100 U/ml), streptomycin (100 mg/ml). For other differentiation methods, 2 μM Retinoic acid (Sigma) was used in this study and Diff 1 medium is the same as mouse spermatogonia stem cell culture medium supplemented with GDNF (R&D systems)^16^.

### Immunofluorescence

For immunocytochemistry, suspension cells were fixed in 4% paraformaldehyde solution and plated on 3-Aminopropyl-Triethoxysilane (APES, 1:50, ZSbio, Beijing, China) treated glass slide. After attachment, the slides were washed three times with PBS (Phosphate-buffered Saline) and permeabilized with PBS containing 0.5% Triton X-100 (Solarbio, Beijing, China) for 10 min. After wash with PBS, the slides were then blocked with PBS supplemented with 10% goat serum (BOSTER, Wuhan, China) and 0.5% Triton X-100 for 30 min at room temperature. For formalin-fixed sections, the sections were deparaffinized in xylene, then rehydrated in grading ethanol. Antigen retrieval was performed in 0.01 M sodium citrate buffer at 96 °C for 10 min. The blocking was carried out with PBS supplemented with 10% goat serum and 30 mg Bovine Serum Albumin (BSA, Solarbio, Beijing, China). After blocking, the slides and sections were incubated with primary antibodies diluted in blocking buffer (10% goat serum, 0.5% Triton X-100, PBS) overnight at 4 °C. Primary antibodies included DAZL (rabbit polyclonal, 1:200), VASA (DDX4, DEAD Box Polypeptide 4, rabbit polyclonal, 1:200), Stella (DPPA3, Developmental Pluripotency Associated 3, rabbit polyclonal, 1:200, Abcam), Βeta1-integrin (ITGB1, Fibronectin Receptor, rabbit polyclonal, 1:1000, Sangon, Shanghai, China), SSEA-1 (Stage-specific Embryonic Antigen-1, mouse monoclonal, 1:200, Millipore, Temecula, USA). After three washes with PBS, the slides and sections were incubated with goat anti-rabbit Ig-CY3/FITC-conjugated secondary antibodies (1:100, Sigma) at 37 °C for 30 min. Finally, the stained slides and sections were mounted with Vecatshield mouting media (Vector, Burlingame, USA) containing Hoechst 33342 (Beyotime, Haimen, China) for nuclei staining.

### Immunofluorescence staining of synaptonemal complexes

For the staining of synaptonemal complexes, cells were lysed by a hypotonic solution and freshly submerged in 1% PFA on glass slides overnight at room temperature. The slides were incubated with 0.04% potoflo for 4 min, then blocked with 1% goat serum at 37 °C for 30 min and incubated with primary antibody against human SCP3 (rabbit polyclonal, 1:200, Novus Biologicals, Littleton, USA) and YH2AX (Phosphorylated Histone H2AX, mouse polyclonal, 1:200, Abcam) for 6–8 h at 37 °C. The slides were further blocked with blocking buffer overnight at 4 °C and incubated with goat anti-rabbit Ig-CY3/FITC-conjugated (1:100, Sigma) secondary antibodies at 37 °C for 2 h and mounted as described above. Linearity fluorescence intensity was analyzed by Image Pro Plus image analysis software 5.1 (Media Cybernetics, Silver Spring, USA).

### Fluorescent *in Situ* Hybridization

Putative 1N populations were fixed with Carnoy’s fixative (1:3 acetic acid: methanol) on APES treated slides for 5 min and then washed with 2×SSC (sodium saline citrate buffer) for 10 min at room temperature. The slides were further incubated in 1% paraformaldehyde (Amersco, Solon, USA) for 10 min and 0.1% NP-40 (Sangon, Shanghai, China) in 2×SSC for 10 min, following by a dehydrated procedure in 80%, 90%, and 100% ethanol series for 2 min each. FISH probes (Chr 16, Chr 18, Abbott Molecular, Illinois, USA) were denatured on slides at 78 °C for 8 min on a hot plate and further hybridized at 37 °C in a moist chamber for 48 h. Finally, the slides were mounted with Vecatshield mouting media (Vector, Burlingame, USA) containing Hoechst 33342 (Beyotime, Haimen, China) for nuclei staining as described above.

### Teratoma formation analysis

About 10 EB like colonies were injected under the kidney capsule of SCID/Beige immunodeficient mice (VITALRIVER, Beijing, China). Kidney capsule injection was carried out as previously described^4^. Briefly, the recipient mouse was anesthetized with 2,2,2-tribromoethanol (Sigma). A skin incision was made on the top of kidney, then a small slit was made on the kidney capsule with a precise forcep. Cells colonies were carefully transplanted under the kidney capsule with a pipet tip. About 3-4 weeks after the surgery, the transplanted tissues were examined and fixed in 4% paraformaldehyde. The sections were further stained with HE for following analysis.

### Karyotyping analysis

Adherent cells were treated with 0.1 μg/ml colchicine for 3.5–4 h at 37 °C, 5% CO_2_, then cells were trypsinized with TypLE Express. Cell pellets were treated with hypotonic solution (0.075 M KCL) for 25 min at 37 °C, then fixed twice in fixative (methanol: glacial acetic acid = 3:1) for 20 min at room temperature. Cell pellets were dropped onto the precooled slides and dried at room temperature. The slides were stained with Giemsa staining solution (Sorlarbio, Beijing, China) for 15 min. Chromosome status was analyzed with chromosome automatic identification image system (Goodline Medical Technology Co., Ltd. Beijing, China).

### DNA content analysis

Single cell pellets were fixed with 70% ethanol overnight at 4 °C, then incubated with PBS supplemented with 50 μg/ml PI (Abcam) and 100 μg/ml RNase A (Beyotime) at 37 °C for 30 min. Analysis was performed with a FACS Calibur flow cytometer (Becton Dicksion, San Jose, USA) and quantification analysis was completed with CellQuest software (Becton Dicksion). 10,000 to 20,000 events were analyzed in each group.

### Statistical Analysis

Variance and statistical comparisons were calculated using GraphPad Prism 5.0 software. Student’s two-tailed t-test was used to determine statistical significance for RT-PCR data. A significant difference was considered at P < 0.05 for all tests.

## Additional Information

**How to cite this article**: Ge, W. *et al*. Differentiation of early germ cells from human skin derived stem cells without exogenous gene integration. *Sci. Rep*. **5**, 13822; doi: 10.1038/srep13822 (2015).

## Supplementary Material

Supplementary Information
