# Supplementary material for: Differentiation of early germ cells from human skin-derived stem cells without exogenous gene integration
Source: Sci Rep. 2015 Sep 8;5:13822. doi: 10.1038/srep13822 (PMC4561906; doi:10.1038/srep13822)
Supplement: Supplementary Information [file srep13822-s1.pdf]

Differentiation of early germ cells from human skin-derived stem cells without exogenous gene integration

Wei Ge[1,2], Hua-Gang Ma[3], Shun-Feng Cheng[1,2], Yuan-Chao Sun[1,2], Li-Lan Sun[3], Xiao-Feng Sun[1,4], Lan Li[1,2], Paul Dyce[4], Julang Li[4], Qing-Hua Shi[5,6,*], Wei Shen[1,2,*]

1 Institute of Reproductive Sciences, Qingdao Agricultural University, Qingdao, Shandong 266109, China;

2 Key Laboratory of Animal Reproduction and Germplasm Enhancement in Universities of Shandong, College of Animal Science and Technology, Qingdao Agricultural University, Qingdao, Shandong 266109, China;

3 Reproductive Center, Weifang City People's Hospital, Weifang, Shandong 261041, China;

4 Department of Animal and Poultry Science, University of Guelph, Guelph, Ontario N1G2W, Canada;

5 Molecular and Cell Genetics Laboratory, The CAS Key Laboratory of Innate Immunity and Chronic Disease, Hefei National Laboratory for Physical Sciences at Microscale, School of Life Sciences, University of Science and Technology of China, Hefei, Anhui 230027, China;

6 Collaborative Innovation Center of Genetics and Development, Fudan University, Shanghai 200433, China

Supplementary Figures

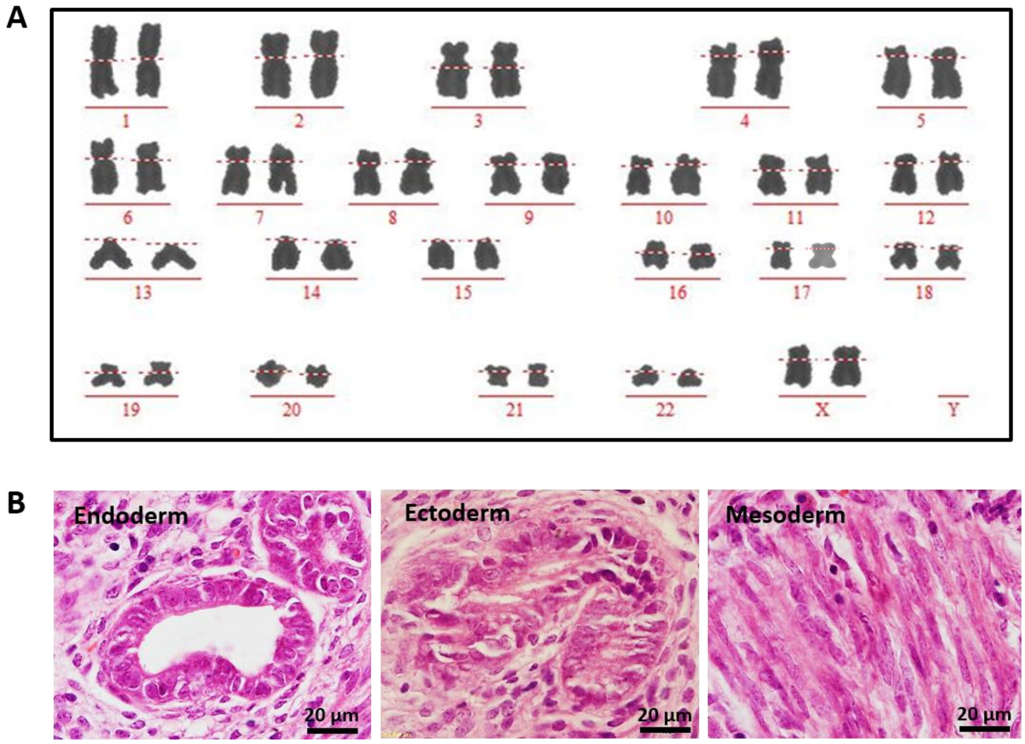

Supplementary Figure 1. Teratoma formation and karyotyping analysis. (A) Karyotyping analysis indicates a normal chromosome status in human fetus skin-derived stem cells (hfSDSCs). (B) Teratoma-like structures were observed when embryoid body (EB)-like colonies were transplanted under the kidney capsule of SCID/Beige mice.

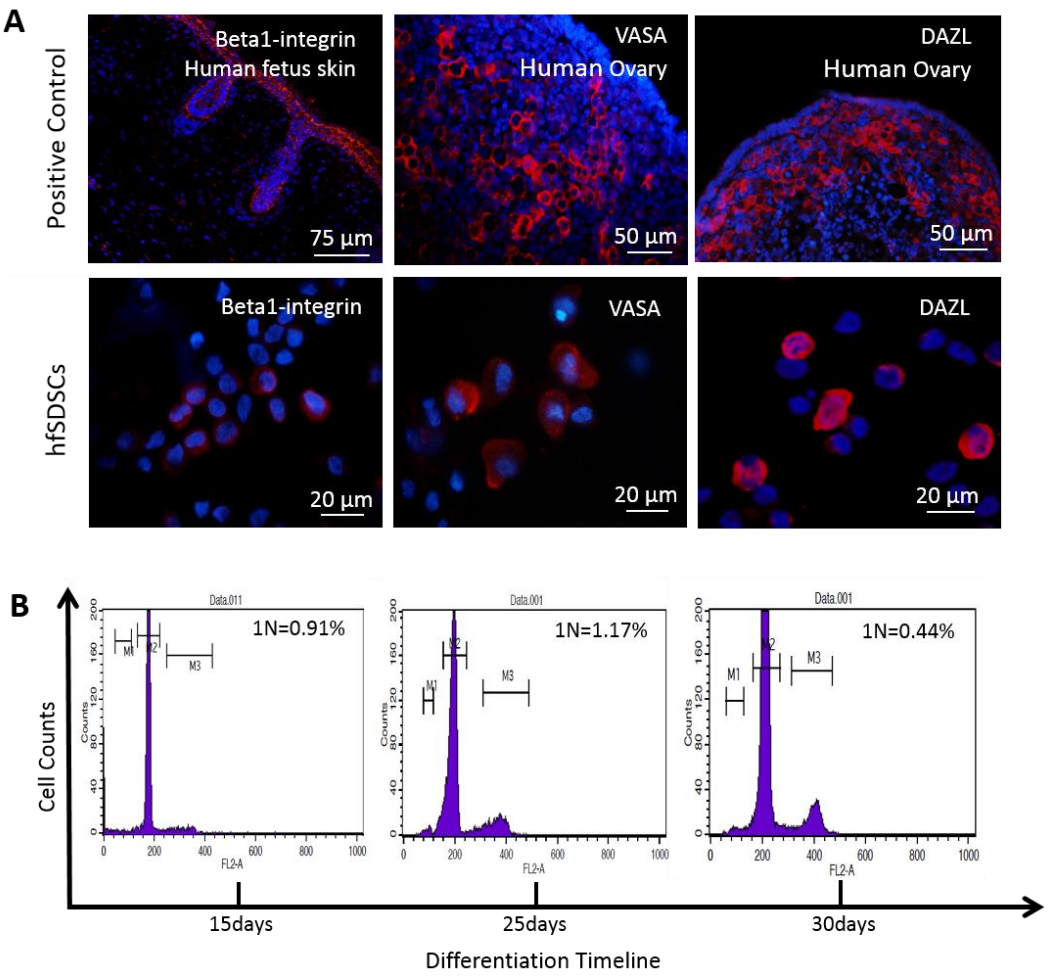

Supplementary Figure 2. Identification of human germ cell-like cells (hGCLCs) and haploid cell formation by immunofluorescence. (A) Expression of Beta1-integrin, DAZL, VASA in hfSDSCs and differentiated hfSDSCs and positive control cells. (B) The dynamic of 1N populations was analyzed by flow cytometry. The highest putative 1N population was observed after 25 days of differentiation.

Supplementary Table 1. DNA content analysis of hfSDSCs differentiated by different methods.

Differentiation days	Differentiation methods	% Haploid	% Diploid	%Tetraploid
18 days	Control	0.21	88.73	5.64
	SDSC+BMP4+RA	0.40	76.04	2.07
	PFF+BMP4+RA	0.18	81.17	4.24
	Diff Media 1	0.21	87.65	1.33
	Diff Media 2	0.91	88.86	4.83
25 days	Control	0.19	88.80	7.60
	SDSC+BMP4+RA	0.44	94.19	4.34
	PFF+BMP4+RA	0.15	92.48	5.09
	Diff Media 1	0.19	95.20	3.58
	Diff Media 2	1.79	80.52	11.51

After 18 days differentiation, two groups showed a little higher percentage of 1N cells (SDSC+BMP4+RA and Diff medium 2) when compared with control (undifferentiated) cells. After 25 days differentiation, a 1.79% (relative value compared with positive control) of 1N population was observed in the cells differentiated with medium 2.

Supplementary Table 2. Primers for quantitative real-time PCR.

Genes	Forward primer	Reverse primer	Genebank Accession number
OCT4	AGTGAGAGGCAACCTGGAGA	GTGAAGTGAGGGCTCCCATA	NM_001285987.1
SOX2	AGAACCCCAAGATGCACAAC	GCTTAGCCTCGTCGATGAAC	NM_003106.3
NANOG	CTGGATTTGTGGGCCTGAA	TGTTTGCCTTTGGGACTGGT	NM_024865.2
DAZL	GACTAATCCAAACACTGAAACTTAT	TACAGTGGTAGTTAACAGCTGAATA	NM_001190811.1
SCP3	AAATCTGGGAAGCCGTCTGT	AACTCCAACTCCTTCCAGCA	NM_001177949.1
VASA	AGCTGGGACATTCAATTCGAC	GTTTGGCGCTGTTCCTTTGAT	NM_001166534.1
AMH	TCCGAGAAGACTTGGACTGG	TCCTCCAGGTGTAGGACCAC	NM_000479.3
GAPDH	GAGTCAACGGATTTGGTCGT	TTGATTTTGGAGGGATCTCG	NM_002046.4
